# Support vector machine deep mining of electronic medical records to predict the prognosis of severe acute myocardial infarction

**DOI:** 10.3389/fphys.2022.991990

**Published:** 2022-09-29

**Authors:** Xingyu Zhou, Xianying Li, Zijun Zhang, Qinrong Han, Huijiao Deng, Yi Jiang, Chunxiao Tang, Lin Yang

**Affiliations:** ^1^ Zhuhai Campus of Zunyi Medical University, Zhuhai, China; ^2^ Key Laboratory of Human-Machine Intelligence-Synergy Systems, Shenzhen Institutes of Advanced Technology, Chinese Academy of Sciences (CAS), Shenzhen, China

**Keywords:** cardiology, electronic medical records, machine learning, support vector machine, ICU

## Abstract

Cardiovascular disease is currently one of the most important diseases causing death in China and the world, and acute myocardial infarction is a major cause of cardiovascular disease. This study provides an analytical technique for predicting the prognosis of patients with severe acute myocardial infarction using a support vector machine (SVM) technique based on information gleaned from electronic medical records in the Medical Information Marketplace for Intensive Care (MIMIC)-III database. The MIMIC-III database provided 4785 electronic medical records data for inclusion in the model development after screening 7070 electronic medical records of patients admitted to the intensive care unit for treatment of acute myocardial infarction. Adopting the APS-III score as the criterion for identifying anticipated risk, the dimensions of data information incorporated into the mathematical model design were found using correlation coefficient matrix heatmaps and ordered logistic analysis. An automated prognostic risk-prediction model was developed using SVM, and the fit was evaluated by 5× cross-validation. We used a grid search method to further optimize the parameters and improve the model fit. The excellent generalization ability of SVM was fully verified by calculating the 95% confidence interval of the area under the receiver operating characteristic curve (AUC) for six algorithms (linear discriminant, tree, Kernel Naive Bayes, RUSBoost, KNN, and SVM). Compared to the remaining five models, its confidence interval was the narrowest with higher fitting accuracy and better performance. The patient prognostic risk prediction model constructed using SVM had a relatively impressive accuracy (92.2%) and AUC value (0.98). In this study, a model was designed for fitting that can maximize the potential information to be gleaned in the electronic medical records data. It was demonstrated that SVM models based on electronic medical records data can offer an effective solution for clinical disease prognostic risk assessment and improved clinical outcomes and have great potential for clinical application in the clinical treatment of myocardial infarction.

## 1 Introduction

Cardiovascular disease is currently one of the most critical diseases causing death and disability worldwide, and it places a significant burden of disease on the population around the world. (Vos et al., 2020). Acute myocardial infarction is ischemic necrosis of myocardial cells and can occur during the natural course of coronary atherosclerosis as an acute coronary syndrome (Reed et al., 2017). As one of the most common cardiovascular diseases, myocardial infarction (MI) is a condition of widespread myocardial necrosis caused by interruption of coronary artery blood supply, resulting in persistent ischemia in the blood supply area, usually complicated by heart failure, heart rupture, and cardiogenic shock. In recent years, the incidence of MI has rapidly increased, and the age composition of MI patients is showing a younger trend, seriously threatening the life and health of human beings. (R. Nasimov et al., 2020). It is estimated that >3 million people suffer an acute ST-segment–elevation MI (STEMI) and >4 million people suffer a non–ST-segment–elevation MI each year. (G. A. Roth et al., 2020). Patients with MI are also at progressively greater risk of re-infarction after discharge from the hospital, and re-infarction or multiple infarctions are a major cause of death in patients with MI (Mal et al., 2019). As a result, it is critical to minimize the mortality rate of MI patients as well as the rate of re-infarction after discharge from the hospital (Nordenskjöld et al., 2019). An accurate evaluation of the prognosis of MI patients may assist health care professionals in devising more appropriate treatment and care plans and in providing more reasonable diagnostic and rehabilitation care in order to enhance the survival rate of MI patients and their quality of life (Than et al., 2019).

The flourishing development of computer technology has played a significant role in enhancing modern health care management, optimizing the allocation of resources, improving efficiency, and reducing medical costs since the third industrial revolution and the gradual maturation of the Internet in the new era. Machine learning algorithms are constantly evolving and have shown effective in medical prediction (Johnson et al., 2021). Machine learning–based predictive models can help less experienced doctors diagnose diseases and improve survival rates by overcoming the drawbacks of relying solely on doctors’ personal subjective experience (He et al., 2022). Prognostic predictive models can also assist health care professionals in developing more reasonable care plans and improving survival rates. Furthermore, electronic medical records (EMRs), which contain medical data, have good guarantee, especially when it comes to using data mining techniques to analyze and process pertinent medical records data (Okamoto et al., 2020). Compared to traditional paper medical records, EMRs can record more information and are easier to keep. As a result, more and more hospitals are choosing to use EMRs to preserve patient-related information. Through appropriate data mining methods, the large amount of information contained in EMRs can be extracted more easily (Ayaad et al., 2019). Machine learning can be used to efficiently use information from electronic medical records in order to achieve a more personalized medicine perspective (Latif et al., 2020).

**FIGURE 1:**
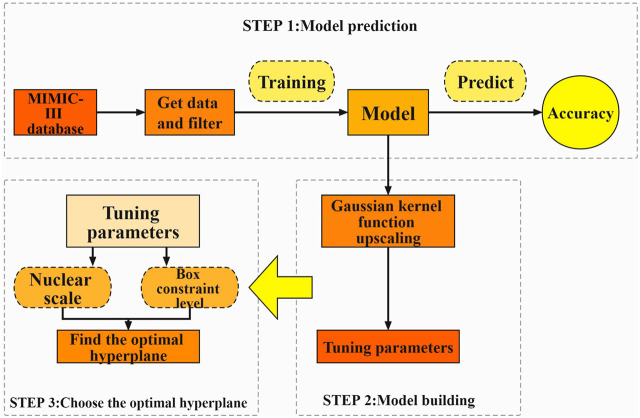
The process of acquiring data from a database and constructing a predictive model.

In this paper, we propose an approach based on a support vector machine (SVM) technique, which can overcome the problems of non-linearity, high dimensionality, and local minima (Hossain et al., 2021) and has a good generalization ability. The support vector machine approach is based on the VC dimensional theory of statistical learning theory and the principle of structural risk minimization, which seeks the best compromise between model complexity and learning ability based on limited sample information in order to obtain the best generalization ability. SVM requires a relatively small number of samples, which is good at coping with the situation of linear indistinguishability of sample data, and also can effectively avoid overfitting to a certain extent. Compared to algorithms such as ordered logstic regression, which are most commonly used in traditional prediction methods, SVMs are structured and stable and have a high generalisation capability. We developed an algorithm that can be used to find out the relationship between the physiological indicators of MI patients and their prognosis using case data screened from the Medical Information Marketplace for Intensive Care (MIMIC)-III database. The model may be used to forecast the prognosis of MI patients, and it can be used in conjunction with the Acute Physiology Score III (APS-III) to precisely assess the prognosis of MI patients (Huang et al., 2021), assuring its dependability. The prediction model constructed in this study can be applied to clinical research. At the same time, however, it can also provide assistance to doctors during diagnosis; may improve their work efficiency; and could alleviate the current situation of medical resources tension in various hospitals, which is of great significance to the treatment and prognosis of MI Figure 1.

The paper is structured as follows. Section 2 of this paper describes the public database required to conduct this experiment and the application of SVM for predictive model building. Section 3 focuses on the evaluation of the model effects in this study. Section 4 of this study synthesizes the current state of research at home and abroad, and provides an objective discussion based on the areas for improvement of this experiment. Section 5 of this study draw a conclusion of the paper and provides future research directions.

## 2 Materials and methods

### 2.1 Data sources

In this study, data analysis and model construction were performed based on sample data from the MIMIC-III database (Wang et al., 2020; Goldberger et al., 2000). In recent years, EMRs have gradually replaced traditional paper charts for recording patient information and have many advantages, such as ease of storage, accuracy of data, and ease of extraction and analysis. MIMIC-III is a large, freely accessible single-center database (Johnson et al., 2016). Developed at the Massachusetts Institute of Technology, it integrates clinical data from patients admitted to the intensive care unit (ICU) at Beth Israel Deaconess Medical Center and is widely used by researchers internationally (Singh and Mayo, 2018; Scherpf et al., 2019).

To protect the security of private patient data, the MIMIC-III database is de-identified using structured data cleansing and date conversion in line with United States Health Insurance Portability and Accountability Act (HIPAA) requirements. All identifiable data element fields listed in HIPAA, such as patient name, phone number, address, and date, are removed throughout the de-identification process for structured data. The removal of protected health information, such as diagnostic reports and medical prescriptions, from strings is completed using a de-identification system based on extensive dictionary look-ups and regular expression patterns. The MIMIC-III database is available as a collection of comma-separated value files and not only has a large sample size and variety of samples but also good reliability (Gentimis et al., 2017).

The researchers responsible for data collection in this project completed a HIPAA-required Protecting Human Research Participants course, signed a data use agreement, and passed the PhysioNet accreditation.

### 2.2 Data acquisition and filtering

To select patients for inclusion, We searched the MIMIC-III database using the keyword “MIMICiii.d_icd_diagnoses where long_title like '%yocardial infarctio%' in the table diagnoses_icd.” We obtained information on all patients admitted to the ICU due to a MI from the MIMIC-III database. We retrieved materialized views MIMICiii.apsiii to obtain a prognostic evaluation of the patient in question. We also retrieved tables of admissions, chart events, laboratory events, microbiology events, and prescriptions to obtain patient-related monitoring data. A total of 7070 relevant data were gathered.

Patients with a high number of missing indicators or EMR data that were incomplete, patients who died while receiving in-hospital care, and patients who suffered a huge number of problems or for whom an MI was just one of many conditions were excluded. A total of 4785 relevant data were finally included.

### 2.3 Data content

Relevant personal information about the patient included length of stay, time treated in the ICU, height, weight, type of health insurance the patient had, and ethnicity. Patient laboratory tests of interest included glucose, triglycerides, N-terminal prenatremic peptide, potassium, platelets, total cholesterol, troponin I, high-density lipoprotein, creatine kinase, troponin T, low-density lipoprotein, C-reactive protein, and creatine kinase isoenzyme. We also considered the following patient pathogenic microbial infections: number of *Staphylococcus aureus* flora, number of *Escherichia coli* flora, and number of *Streptococcus pneumoniae* flora.

Finally, we recorded the total dose of different drugs administered during treatment, including aspirin, heparin, atorvastatin, *mycoplasma*, and nitroglycerin. The prognostic model score for patients was the APS-III score.

### 2.4 Details of the proprietary software

In this study, the software used to construct the model was MatLab (R2021a 9.10.0.1602886; The MathWorks, Inc. Natick, MA, United States). To describe the correlation between features, a correlation coefficient matrix heatmap was drawn using the R language (version 4.1.3; The R Foundation for Statistical Computing, Vienna, Austria).

### 2.5 Theory/calculation

#### 2.5.1 Prognosis evaluation method

The concept of objective evaluation of critically ill patients’ conditions has become widely accepted by clinical workers alike as an important tool in their daily work, and various scores were widely used in clinical applications of this study. In the MIMIC-III database, in addition to the APS-III scale (Knaus et al., 1991) there exist such scales as the Oxford Acute Severity of Illness Score (OASIS) (Holland and Moss, 2017), Sepsis-related Organ Failure Assessment (SOFA) (Lambden et al., 2019), Logistic Organ Dysfunction Score (LODS) (Marshall, 2020), Scale for Assessing Positive Symptoms (SAPS) (Le Gall et al., 1993), and many other scales used in critical care medicine.

Compared to the above-mentioned scales, the APS-III scale—as one of the widely used tools for critical illness assessment—has been shown in many studies to be significantly associated with patient survival evaluation (Pathmanathan, 2005). The APS-III scale was designed to reflect individual differences in acute physiological status, age, and chronic disease status (Godinjak, 2016; Sadaka et al., 2017). Excellent predictive results have been achieved in evaluating the effectiveness of medical measures, predicting patient prognosis, making predictions about the risk of death in individuals and groups, classifying patients according to their condition, and comparing treatment outcomes (Moreno and Nassar Júnior, 2017).

**FIGURE 2:**
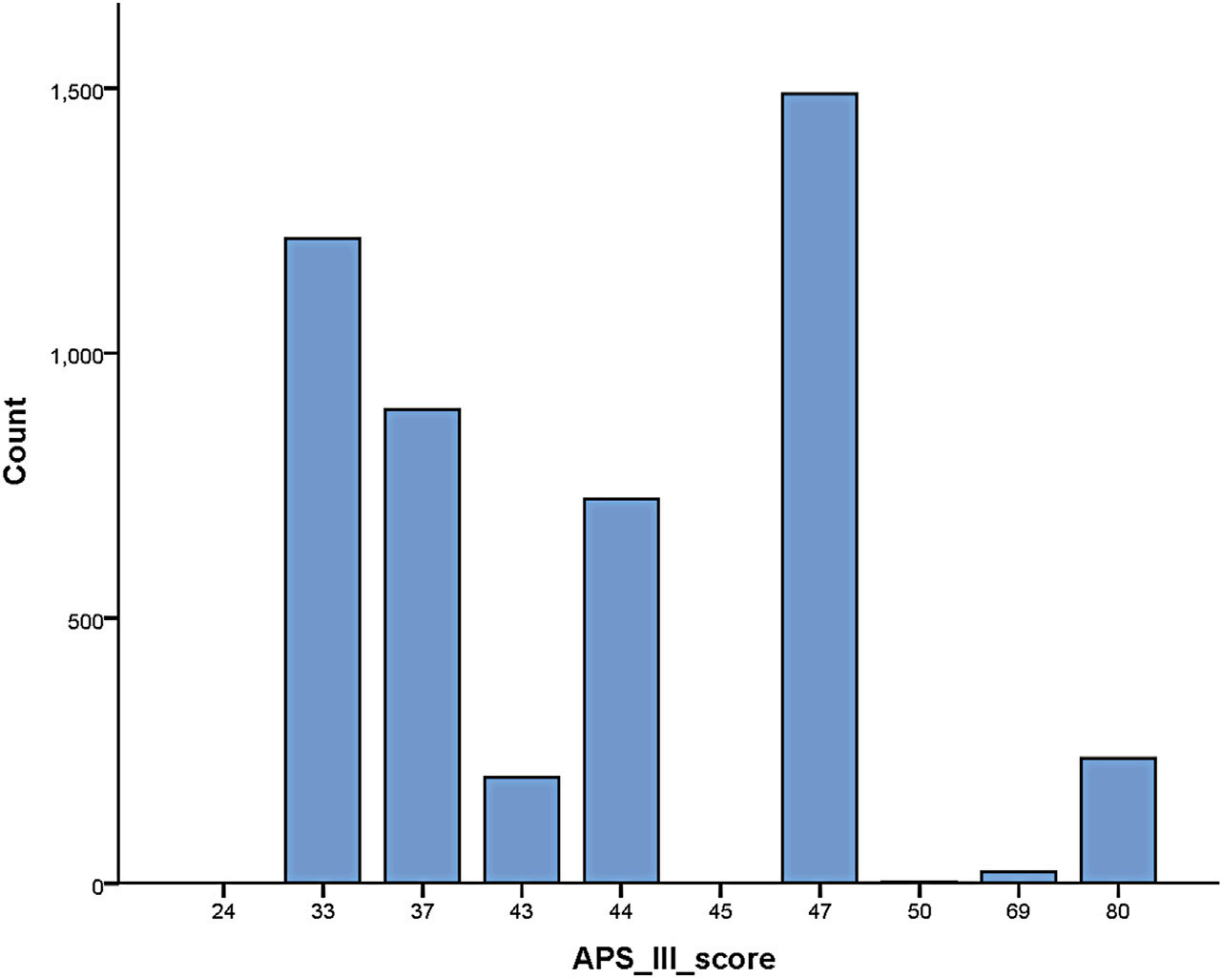
APS-III scale scores for patients included in the study.

The APS-III scale has been widely used in the medical community as an important tool for predicting the risk of death prediction in ICU patients. In a recent study on prognosis prediction of ICU patients (Zhang et al., 2022), the results showed that the independent receiver operating characteristic curve (ROC) curve results of the APS-III scale were superior compared to those of the SAPS-II, LODS, OASIS, and SOFA scales, indicating that the former has a more promising accuracy in the prognosis prediction of critically ill patients. Thus, the results of the APS-III scale were used to evaluate the prognosis of patients in this study Figure 2.

#### 2.5.2 Feature extraction and analysis

Redundant or less relevant variable features often exist in multidimensional data, which affects the accuracy of machine learning output (Ho et al., 2019). Feature selection can solve this drawback, reduce the burden of machine learning, and improve the generalization performance, prediction performance and operational efficiency of the algorithm (Chandrashekar and Sahin, 2014).

Correlation analysis between features and APS-III can select features that are meaningful for classification prediction results from all features of sample data, so as to exclude the interference of chance factors in the data. Therefore, in this paper, the correlation coefficients between features and APS-III are calculated and the heat map of the correlation coefficient matrix is drawn to investigate whether there is a correlation between features and APS-III, and the direction and magnitude of the correlation relationship (Haarman et al., 2015).

**FIGURE 3:**
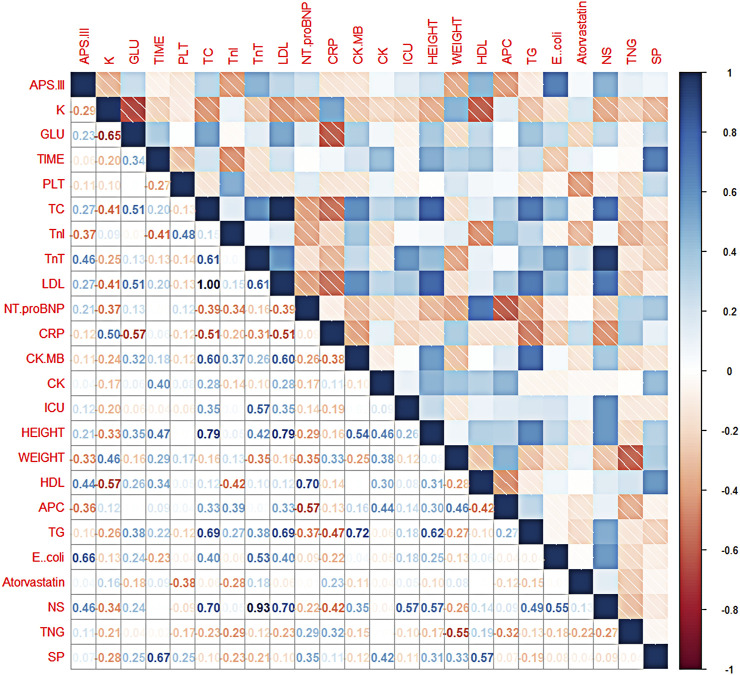
Thermalmatrix diagram of correlation coefficients for each feature. Description of the abbreviations in Figure 3: K (blood potassium), GLU (blood glucose), TIME (length of hospital stay), PLT (platelets), TC (total cholesterol), TnI (troponin I), TnT (troponin T), LDL (low-density lipoprotein), Nt. proBNP (N-terminal prenatriuretic peptide), CRP (C-reactive protein), CK. MB (creatine kinase isoenzyme), CK (creatine kinase), ICU (patient’s time in ICU), HEIGHT (patient’s height), WEIGHT (patient’s weight), HDL (high-density lipoprotein), APC (total aspirin dose), TG (triglycerides), E.*coli* (number of *Escherichia coli* flora), Atorvastatin (total atorvastatin dose), NS (total bacteriocin does), TNG (total nitroglycerin dose), SP (*Streptococcus pneumoniae*).

In this study, first the corrplot package was installed and imported in R language and a dataset in csv format was loaded, then the calculation of the matrix of correlation coefficients between all features was started and two decimal places were retained, and finally the matrix of correlation coefficients was plotted using the corrplot package to create a heat map of the correlation coefficient matrix for all features (as in Figure 3). In the correlation coefficient matrix heatmap, each number represents the correlation coefficient between the corresponding features, and the color shades of the corresponding squares also symbolize the size of the correlation coefficient, i.e., the darker the color, the larger the correlation coefficient, and vice versa. The color of the squares is related to the direction of correlation, with blue representing a positive correlation and red representing a negative correlation. In this study, APS-III was used as a predictor of patient prognosis evaluation. The correlation coefficients between “Length of hospital stay”, “Platelets”, “C-reactive protein”, “Creatine kinase isoenzyme”, “Creatine kinase”, “Length of stay in ICU”, “Triglycerides”, “Total dose of atorvastatin “, “total nitroglycerin dose”, “*Streptococcus pneumoniae*” and APS-III scores were all low, all <0.2. These indicators were removed in the later model construction. Indicators included in the final model construction were: blood potassium, blood glucose, total cholesterol, troponin I, troponin T, HDL, LDL, N-terminal prenatremic peptide, height, weight, E. coli, total aspirin dose, total *mycoplasma* dose.

#### 2.5.3 SVM

**FIGURE 4:**
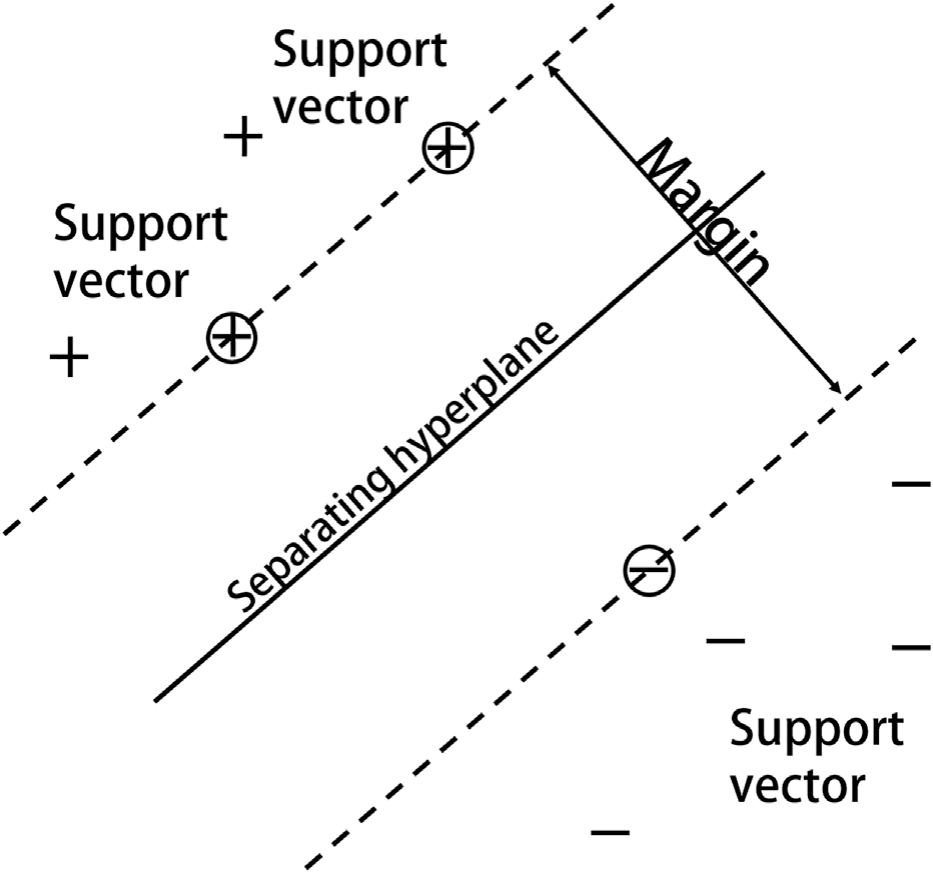
SVM schematic.

Based on statistical learning theory and the notion of structural risk minimization, Vapnik and others at AT&T Bell Labs introduced SVM for classification and regression investigations (Vapnik, 2000). SVM classifies data by determining the optimum hyperplane for successfully separating a data point class from another (Figure 4). By non-linearly mapping the input space to the high-dimensional feature space, the kernel function can make classification more convenient and effective. The Gaussian radial basis kernel function SVM classification ability is significantly superior to other approaches in the face of non-linear classification issues (Liu et al., 2012), and using SVM on this basis can provide more scientifically accurate results.

The kernel parameter ( γ ) is the only variable parameter in the space mapped by the Radial Basis Function kernel function, i.e., the value of γ directly influences the distribution of sample data in the kernel space; hence, the optimal value of γ substantially affects the model fit accuracy (Padierna et al., 2018).

The penalty term C is used to limit the model’s complexity and accuracy, i.e., to adjust the learning machine’s confidence range to the empirical risk in a specific feature subspace, so that the learning machine can generalize as well as possible. The greater the C value, the better the model fits, although this does not guarantee generalization (Tharwat, 2019). In each subspace, there is only 1 optimal penalty term for constraining the entire model; nevertheless, in order to attain high accuracy, this single element must be examined in isolation.

**FIGURE 5:**
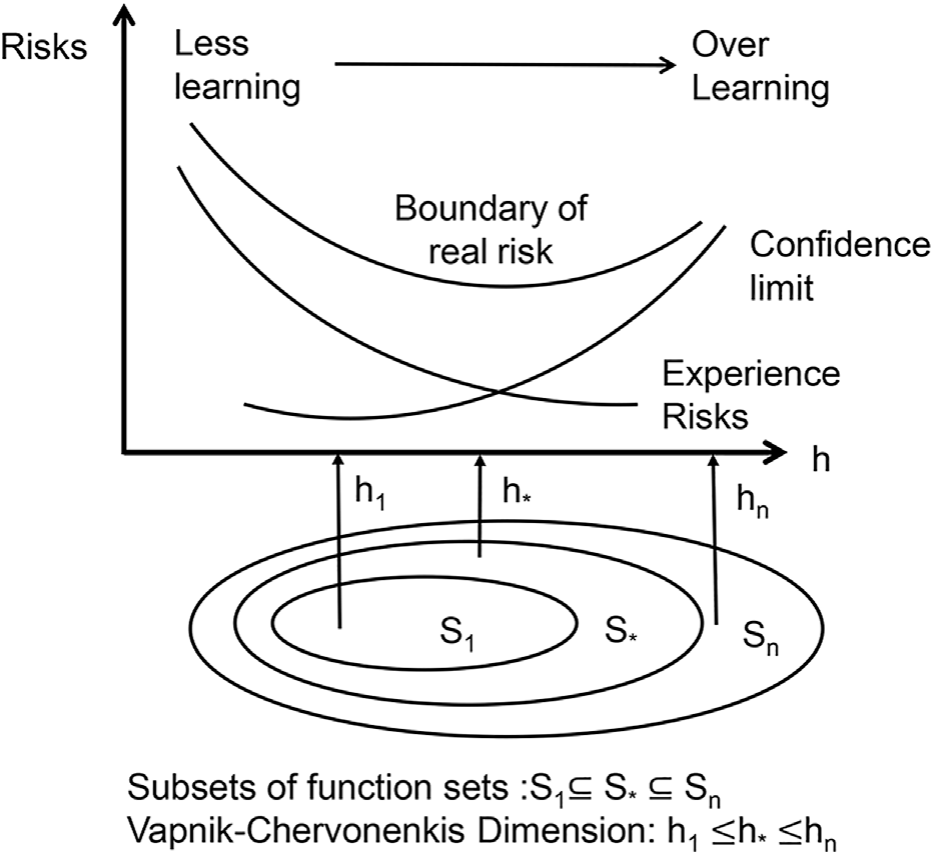
Schematic diagram of the principle of structural risk minimization.

The basis of SVMs is the structural risk minimization (SRM) principle (Shawe-Taylor et al., 1998). The core of the SRM principle is to reduce the complexity of the learning machine, that is the Vapnik-Chervonenkis dimension (VC dimension), while maintaining classification accuracy (experience risks), which allows the expected risk of the learning machine to be controlled over the entire sample set (as in Figure 5). Because the SRM principle’s premise is for a specific subspace in the feature space and the data contain different divisions in the non-stop subspace, there are different optimal SVM algorithms in different subspaces; therefore, the SVM kernel parameters and the penalty term C must be optimized at the same time. In this study, we used a grid search approach to discover the optimum combination of C and hyperplane, then produced the best-fitting SVM model.

#### 2.5.4 Algorithm steps

SVM is a new type of machine learning algorithm. The ideal hyperplane fulfills the following inequality for a given sample set of variables ( $x_{i}$ , $y_{i}$ ) i = 1,2,…,n. In the case of the input variable $x_{i}\in R^{d}$ and the output variable $y_{i}\in \left\{-1,1\right\}$ , φ(⋅) is a nonlinear function, the optimal hyperplane satisfies the following inequality:

$$y_{i}\left[w^{T}\varphi \left(x_{i}\right)+b\right]\geq 1-\xi_{i}$$ (1)

where $w^{T}$ is a multidimensional vector, b is a constant, and $\xi_{i}$ is a slack variable related to the classification error. To maximize the distance between the 2 categories, the above inequality can be rewritten as:

$$min\left[\frac{1}{2}{\left|w\right|}^{2}+\text{C}\sum_{i=1}^{n}\xi_{i}\right]$$ (2)

where C is a penalty term that adjusts the relaxation variable $\xi_{i}$ to determine the classification error and also the classification interval $\frac{1}{2}{\left|w\right|}^{2}$ . For non-linear indistinguishable sample points, a kernel function is introduced to map the sample points to a higher dimensional space, thus achieving an effective classification of the sample points.

The radial basis kernel function is expressed as follows:

$$K\left(x,x^{\prime }\right)=exp\left(-\frac{{\left\|x-x^{\prime }\right\|}^{2}}{2\sigma^{2}}\right)=exp\left(-\gamma {\left\|x-x^{\prime }\right\|}^{2}\right)$$ (3)

where the radial basis function (RBF)kernels of two samples, x and $x^{\prime }$ , are represented as eigenvectors in some input space; σ is the bandwidth of the Gaussian radial basis kernel function; γ is the parameter of the Gaussian radial basis kernel function; and exp denotes the exponential function with natural constant e as the base. Also, γ takes the general values $\gamma =\left\{2^{-15},2^{-14},\ldots ,2^{15}\right\}$ . In this study, by the grid search method, γ is substituted sequentially into the following equation:

$$D_{\left(c_{1},c_{2}\right)}={\mathrm{\|m}}_{1}-m_{2}^{2}\|=\frac{1}{l_{1}^{2}}\sum_{i=1}^{l_{1}}\sum_{j=1}^{l_{1}}\exp\left(-\gamma {\left\|x_{i}^{\left(1\right)}-x_{j}^{\left(1\right)}\right\|}^{2}\right)+\frac{1}{l_{2}^{2}}\sum_{i=1}^{l_{2}}\sum_{j=1}^{l_{2}}\exp\left(-\gamma {\left\|x_{i}^{\left(2\right)}-x_{j}^{\left(2\right)}\right\|}^{2}\right)-\frac{1}{l_{1}l_{2}}\sum_{i=1}^{l_{1}}\sum_{j=1}^{l_{2}}\exp\left(-\gamma {\left\|x_{i}^{\left(1\right)}-x_{j}^{\left(2\right)}\right\|}^{2}\right)$$ (4)

The grid search method is an exhaustive search method that divides all of the parameters γ and C to be searched into a grid of the same length in a given space, traverses each grid, and then writes a program to optimize the SVM model using MatLab to find the best combination of parameters with the smallest mean square error (Fayed and Atiya, 2019). Compared to the traditional exhaustive search method, this method is more accurate and easier to use when looking for the best combination of parameters. This work involves the use of cross-validation to evaluate the classification accuracy of the model created for each parameter combination in order to improve its fitting effect and acquire a better generalization capability.

In Formula (4), $D_{\left(c_{1},c_{2}\right)}$ is the distance measure obtained from measure learning. The optimal kernel parameter is that which corresponds to the largest kernel space mean distance where $m_{1}$ and $m_{2}$ are the feature space centroid vectors for the first and second classes of data, respectively. The formula for the particular derivative is as follows:

$$m_{1}=\frac{1}{l_{1}}\sum_{i=1}^{l_{1}}\text{Φ}\left(x_{i}^{\left(1\right)}\right)$$ (5)

$$m_{2}=\frac{1}{l_{2}}\sum_{i=1}^{l_{2}}\text{Φ}\left(x_{i}^{\left(2\right)}\right)$$ (6)

In conclusion, the optimum parameter combination for the following tests in this study is a box constraint level of 30 and a kernel scale value of 250. The soft interval size in SVM, which is stated as the penalty term (C) in RBF, is connected to the box constraint. The lower the value, the lower the penalty, which impacts the model fit, and the higher the value, the higher the penalty, which reduces the model accuracy. It is simple to know that $\text{KernelScale}=\sqrt{\frac{1}{\gamma }}$ because $\text{KernelScale}=\sqrt{2}\sigma$ ( σ is the bandwidth) and Eq. 3 are combined. As a result, the kernel parameters dictate the value of the kernel scale, which affects the model’s accuracy.

## 3 Results

By plotting the correlation coefficient matrix heatmap of all features, the researchers removed indicators with low correlations with APS-III scores. After ensuring the relevance of the data, validation of the accuracy of the model is equally essential. The 5× cross-validation method was used to verify the model accuracy in predicting the prognosis of acute MI disease. The samples in the dataset were separated into five groups, with four groups used to train the model and one used to test it. Five rounds of the above experiments were run, and the average value of the five training results was used to determine the model’s accuracy.

**FIGURE 6:**
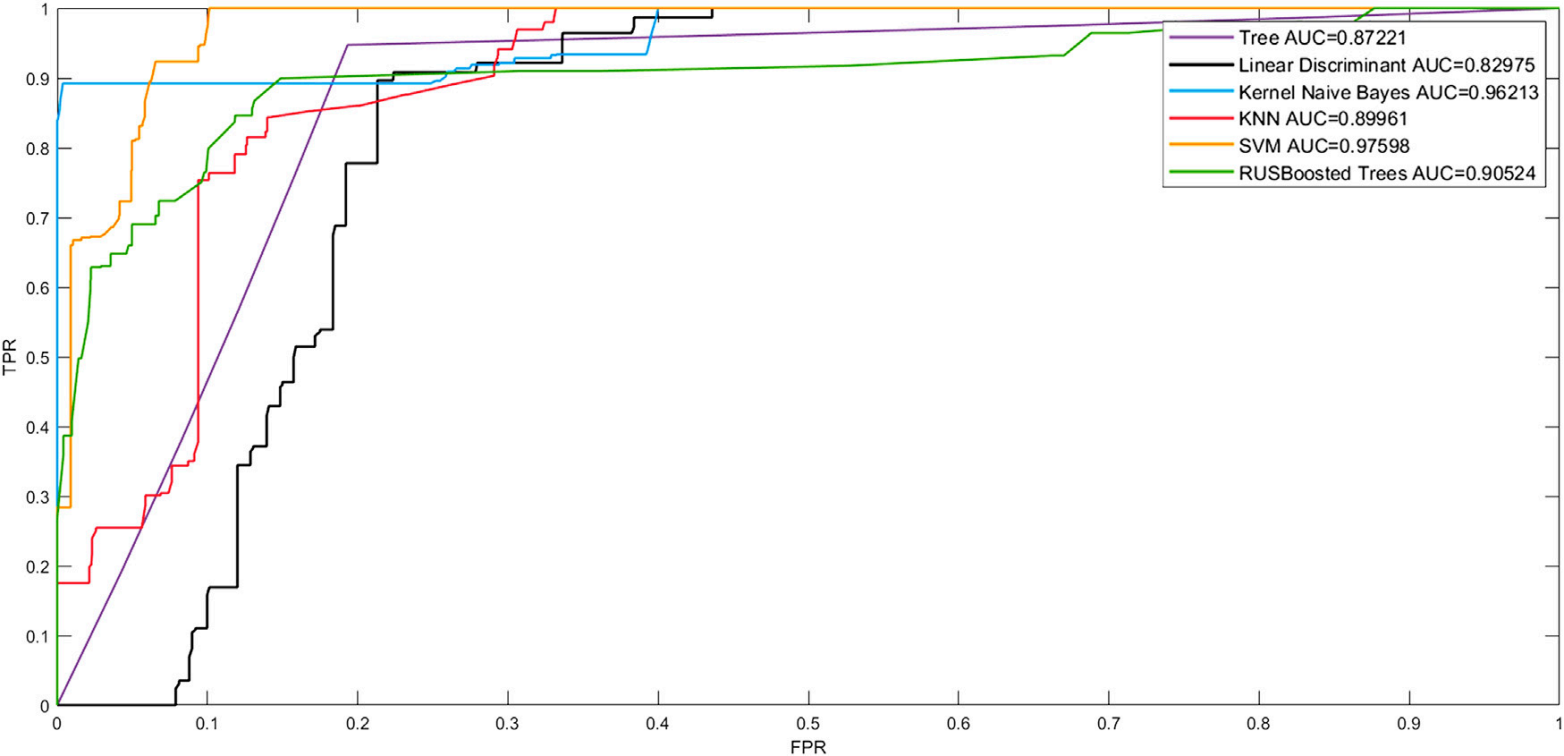
ROC curves for the models.

The Receiver Operating Character curve (ROC curve), with the false-positive rate (FPR) as the horizontal axis and the true-positive rate (TPR) as the vertical axis, is a commonly used model evaluation metric in the medical field. The area under the ROC curve, or AUC (Area Under ROC Curve), is a visual representation of the model’s performance. The number of AUCs is a measure of the model’s overall quality, with a greater AUC indicating better model performance. To verify the effectiveness of the model fit in this study, we plotted the linear discriminant, support Vector Machine (SVM) tree, Kernel Naive Bayes, random undersampling boost (RUSBoost), and K- NearestNeighbor (KNN) ROC curves to show the performance of the currently selected training classifiers. As shown in Figure 6, in terms of model classification performance, the SVM algorithm obtained the ROC curve closest to the upper left corner and the largest AUC with an AUC of 0.97598. Kernel Naive Bayes has the second highest AUC value of 0.96213, which proves that the algorithm is also able to meet certain clinical needs in terms of model fitting. However, the best performing model was still the prognostic prediction model constructed by SVM.

For data with a large sample content, the AUC approximates a normal distribution, so the 95% confidence interval (CI) for the AUC can be calculated as described in the CI of the sampling distribution.

The CI is equal to $C\;\pm \;se-z_{crit}$ , where $z_{crit}$ is the two-tailed critical value of the standard normal distribution.

$$se=\sqrt{\frac{q_{0}+\left(n_{1}-1\right)q_{1}+\left(n_{2}-1\right)q_{2}}{n_{1}n_{2}}}$$

$n_{1}$ and $n_{2}$ are the sizes of the 2 samples, respectively.

$$q_{0}=AUC\left(1-AUC\right)\;q_{1}=\frac{AUC}{2-AUC}-AUC^{2}q_{2}=\frac{2AUC^{2}}{1+AUC}-AUC^{2}$$

The DeLong test is a relatively common method of AUC significance test. The principle is as follows. Taking two different models as an example, let the two AUCs be A_1_ and A_2_ respectively.

1 First calculate the difference between the two AUC values.

$$\theta =A_{1}-A_{2}$$

2 Calculate the variances var (A_1_) and var (A_2_) of A_1_ and A_2_, and the covariance cov (A_1_, A_2_) of the two.
3 Calculate the Z-value

$$Z=\frac{\theta }{var\left(A_{1}\right)+var\left(A_{2}\right)-2cov\left(A_{1},A_{2}\right)}$$

4 Finally, take the Z-value distribution as a normal distribution, do a significance test, and get the P value. If the p value is less than 0.05, it means that there is a significant difference between the two AUCs, which is statistically significant, otherwise, it is not significant.

**FIGURE 7:**
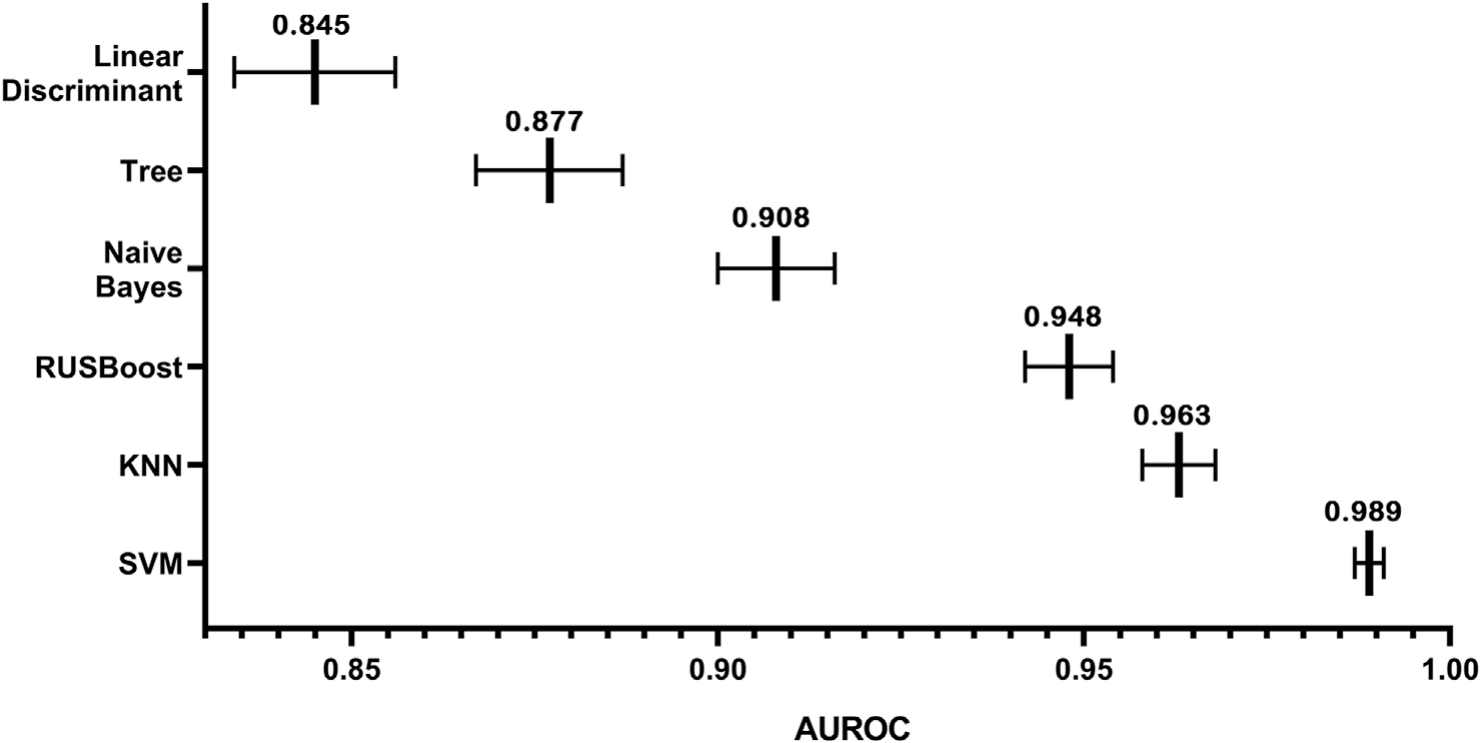
Visual overview of the AUC and 95% CI values for each model.

In this study, we had a total sample of 4785 cases and used 5× cross-validation to calculate the values of AUC for linear discriminant, tree, Kernel Naive Bayes, RUSBoost, KNN, and SVM, as shown in Figure 7 Using this method, it can be concluded that the algorithm with the highest value of AUC and the narrowest CI is the SVM algorithm. As shown in Table 1, the DeLong test was performed on the ROC of SVM and the ROC of other algorithms, and the obtained *p*-values were all less than 0.05, indicating that there was a significant difference between the AUC of the SVM algorithm and the use of other algorithms, which was statistically significant, further indicating that the model built using the SVM algorithm has better accuracy. The AUC values for linear discriminant, tree, Kernel Naive Bayes, RUSBoost, KNN, and SVM increased sequentially, indicating that the predictive ability of each model increased sequentially and the CI decreased sequentially, which implies that there is a decreasing uncertainty in the prognostic effect of each model in predicting patients with MI. Therefore, we can conclude that when using the existing dataset for prediction model construction, the prediction model constructed by SVM has a more promising fit than the remaining five algorithms.

**TABLE 1:** Conclusion of DeLong test of SVM with other five classifiers.

Classifier	Z-value	*p*-value
KNN	16.536	<2.2e-16
RUBoost	34.198	<2.2e-16
Naïve Bayes	10.448	<2.2e-16
Tree	9.0918	<2.2e-16
Liner Discriminant	28.143	<2.2e-16

## 4 Discussion

The scale-based assessment of patient condition is one of the foundations of our project, but this study has considerable advantages over scale-based assessment. Compared to the current predictive model, scales are time-consuming and difficult to obtain when used alone and can even more difficult to obtain if a patient has specific conditions, such as hearing or vision loss or speech impairment, making it difficult for health care professionals to accurately determine a patient’s condition in a timely manner (Arnetz et al., 2008). In this study, using the obtained scales as the basis for the prognosis of the model can largely reduce the process of obtaining the patient’s scale scores and can present the findings in a dynamic manner to obtain more accurate and rapid predictions, which can reduce the workload of the clinical staff and help physicians to accurately determine the progress of the disease, thus assisting them in making individualized adjustments to the treatment plan. In other words, the present system will help doctors to make personalized adjustments to treatment plans.

In this study, the biomarkers of our prognostic prediction model are widely used clinically. This may ensure the general applicability of our study results and provides a useful adjunct for clinical treatment. Due to the combination of machine learning and medicine, the large, complex, and multidimensional datasets present in EMRs can be analyzed. For instance, Lee et al. (2021) developed a deep learning–based method used to screen fundus abnormalities in patients with high specificity and sensitivity. In addition, Zhao et al., using artificial intelligence–based algorithms combined with 12-lead electrocardiogram (ECG) data, developed an accurate early warning system based on ECG data, and the sensitivity of the model was 99%. They also proposed a wearable ECG vest, and smartphones and real-time warning systems coupled with an automatic diagnosis will greatly improve the diagnosis rate for STEMI patients and reduce patient delay times (Zhao et al., 2020).

In recent years, in the context of the era of big data in healthcare, with the development of artificial intelligence technology, more and more researchers are using machine learning, such as K-NearestNeighbor (KNN), the Bayes algorithm, and the decision tree to build predictive models. The KNN method is a lazy learning method that uses instances to discover the K training dataset that is the most similar to the unknown data. Its sample pool size is necessary, which severely restricts its practical application if the sample set is complex or if training samples are not available (Zhang and Zhou, 2007). The Bayesian classification algorithm is a probabilistic statistics-based classification method that considers all qualities and theoretically yields the best solution with the least amount of error. However, the accuracy of its classification may be affected because Bayes’ theorem presupposes that the effect of an attribute value on a given class is independent of the values of other attributes, which is frequently false (Manino et al., 2019). A decision tree is a tree-like instance-based inductive classification algorithm that can classify and predict at the same time. However, due to its extreme bifurcation, it is prone to overfitting, and the error can rapidly increase when there are too many categories (Myles et al., 2004). In contrast, SVM, as a supervised learning algorithm, has a rigorous mathematical theoretical support, possesses good interpretability, and does not rely on statistical methods to some extent. SVM’s final decision function is determined by only a few vectors, has no significant correlation with sample space dimensionality, and can identify support vectors that are critical to the project (Noble, 2006). SVM has been widely used by the international medical community in recent years to solve the classification regression aspects of biological data, such as in the prognosis prediction of patients with serious diseases like laryngeal cancer (Chen et al., 2007), prostate cancer (Çınar et al., 2009), hepatocellular carcinoma (Ali et al., 2021), and renal cell tumors (Giulietti et al., 2021).

Past studies (Than et al., 2019; Doudesis et al., 2022) used a single physiological condition as an indicator to assess the prognosis of patients or their mortality. However, we believe that the underlying individual circumstances of the patient, as well as their status in society and ethnicity, also largely influence the progression of their disease (Khraim and Carey, 2009). In addition, the different treatment strategies received by different patients during their in-hospital stay also have a significant impact on the prognosis (Anderson and Morrow, 2017). Thus, in this study, we not only included the physical condition of patients in the screening of characteristics but also their health insurance status, height, weight, age, ethnicity, and even the length of time they were treated for in the ICU and the dosage of the injected drugs. The inclusion of multiple dimensions of the patient’s condition inevitably allows for a more comprehensive perspective on the progression of said condition. The collection of these characteristics largely facilitates the completeness of the model and allows for an accurate evaluation of the patient from multiple perspectives, which in turn leads to more valid predictive conclusions.

In this study, the data used in this study came from Massachusetts General Hospital in the United States, which limits the model’s applicability. More localization is needed to improve the model’s applicability so that it can help health care professionals make more accurate predictions about the prognosis of MI patients in the future, assisting in the development of appropriate treatment and care plans and improving the prognosis.

## 5 Conclusion

We retrieved EMRs from the MIMIC-III database and analyzed them with R to discover that 13 markers, such as blood potassium, blood glucose, and total cholesterol, have a strong link with the prognosis of MI patients. A patient prognostic model was built by comparing plain Bayesian, KNN, linear discriminant, RUSBoost trees, and SVM algorithms, and the prognostic model based on the SVM algorithm was found to have a good fit, with an accuracy rate of 92.2% and an AUC of 0.989, demonstrating that the model still has a certain (necessarily higher) accuracy and conviction compared to other algorithms. SVM feature extraction from EMR data enhances prediction accuracy, and this technology is universally applicable, allowing it to be used for prognostic prediction of different diseases.

## Data Availability

The datasets presented in this study can be found in online repositories. The names of the repository/repositories and accession number(s) can be found below: https://mimic.physionet.org.

## References

[B1] Ali L. Wajahat I. Amiri Golilarz N. Keshtkar F. Bukhari S. A. C. (2021). LDA–GA–SVM: Improved hepatocellular carcinoma prediction through dimensionality reduction and genetically optimized support vector machine. Neural comput. Appl. 33, 2783–2792. 10.1007/s00521-020-05157-2

[B2] Anderson J. L. Morrow D. A. (2017). Acute myocardial infarction. N. Engl. J. Med. 376, 2053–2064. 10.1056/NEJMra1606915 28538121

[B3] Arnetz J. E. Winblad U. Arnetz B. B. Höglund A. T. (2008). Physicians’ and nurses’ perceptions of patient involvement in myocardial infarction care. Eur. J. Cardiovasc. Nurs. 7, 113–120. 10.1016/j.ejcnurse.2007.05.005 17581793

[B4] Ayaad O. Alloubani A. ALhajaa E. A. Farhan M. Abuseif S. Al Hroub A. (2019). The role of electronic medical records in improving the quality of health care services: comparative study. Int. J. Med. Inf. 127, 63–67. 10.1016/j.ijmedinf.2019.04.014 31128833

[B5] Chandrashekar G. Sahin F. (2014). A survey on feature selection methods. Comput. Electr. Eng. 40, 16–28. 10.1016/j.compeleceng.2013.11.024

[B6] Chen W. Peng C. Zhu X. Wan B. Wei D. (2007). “SVM-based identification of pathological voices,” in 2007 29th annual international conference of the IEEE engineering in medicine and biology society (Lyon, France: IEEE), 3786–3789. 10.1109/IEMBS.2007.4353156 18002822

[B7] Çınar M. Engin M. Engin E. Z. Ziya Ateşçi Y. (2009). Early prostate cancer diagnosis by using artificial neural networks and support vector machines. Expert Syst. Appl. 36, 6357–6361. 10.1016/j.eswa.2008.08.010

[B8] Doudesis D. Lee K. K. Yang J. Wereski R. Shah A. S. V. Tsanas A. (2022). Validation of the myocardial-ischaemic-injury-index machine learning algorithm to guide the diagnosis of myocardial infarction in a heterogenous population: a prespecified exploratory analysis. Lancet. Digit. Health 4, e300–e308. 10.1016/S2589-7500(22)00025-5– 35461689 PMC9052331

[B9] Fayed H. A. Atiya A. F. (2019). Speed up grid-search for parameter selection of support vector machines. Appl. Soft Comput. 80, 202–210. 10.1016/j.asoc.2019.03.037

[B52] Gentimis T. Alnaser A. J. Durante A. Cook K. Steele R. (2017). “Predicting Hospital Length of Stay Using Neural Networks on MIMIC III Data,” in 2017 IEEE 15th Intl Conf on Dependable, Autonomic and Secure Computing, 15th Intl Conf on Pervasive Intelligence and Computing, 3rd Intl Conf on Big Data Intelligence and Computing and Cyber Science and Technology Congress (DASC/PiCom/DataCom/CyberSciTech), (Orlando, FL: IEEE), 1194–1201. 10.1109/DASC-PICom-DataCom-CyberSciTec.2017.191

[B10] Giulietti M. Cecati M. Sabanovic B. Scirè A. Cimadamore A. Santoni M. (2021). The role of artificial intelligence in the diagnosis and prognosis of renal cell tumors. Diagnostics 11, 206. 10.3390/diagnostics11020206 33573278 PMC7912267

[B11] Godinjak A. G. Iglica A. Rama A. Tancica I. Jusufovic S. Ajanovic A. (2016). Predictive value of SAPS II and Apache II scoring systems for patient outcome in a medical intensive care unit. Acta Med. Acad. 45, 97–103. 10.5644/ama2006-124.165 28000485

[B50] Goldberger A. L. Amaral L. A. N. Glass L. Hausdorff J. M. Ivanov P. Ch. Mark R. G. (2000). PhysioBank, physio toolkit, and physionet: Components of a new research resource for complex physiologic signals. Circulation 101. 10.1161/01.CIR.101.23.e215 10851218

[B12] Haarman B. C. M. Benno) Riemersma-Van der Lek R. F. Nolen W. A. Mendes R. Drexhage H. A. (2015). Feature-expression heat maps—a new visual method to explore complex associations between two variable sets. J. Biomed. Inf. 53, 156–161. 10.1016/j.jbi.2014.10.003 25445923

[B13] He Z. Yuan S. Zhao J. Du B. Yuan Z. Alhudhaif A. (2022). A novel myocardial infarction localization method using multi-branch DenseNet and spatial matching-based active semi-supervised learning. Inf. Sci. 606, 649–668. 10.1016/j.ins.2022.05.070

[B14] Ho D. S. W. Schierding W. Wake M. Saffery R. O’Sullivan J. (2019). Machine learning SNP based prediction for precision medicine. Front. Genet. 10, 267. 10.3389/fgene.2019.00267 30972108 PMC6445847

[B15] Holland E. M. Moss T. J. (2017). Acute noncardiovascular illness in the cardiac intensive care unit. J. Am. Coll. Cardiol. 69, 1999–2007. 10.1016/j.jacc.2017.02.033 28427574

[B16] Hossain M. E. Khan A. Moni M. A. Uddin S. (2021). Use of electronic health data for disease prediction: a comprehensive literature review. IEEE/ACM Trans. Comput. Biol. Bioinform. 18, 745–758. 10.1109/TCBB.2019.2937862 31478869

[B17] Huang W.-C. Xie H.-J. Fan H.-T. Yan M.-H. Hong Y.-C. (2021). Comparison of prognosis predictive value of 4 disease severity scoring systems in patients with acute respiratory failure in intensive care unit: a STROBE report. Medicine 100, e27380. 10.1097/MD.0000000000027380 34596157 PMC8483864

[B18] Johnson A. E. W. Pollard T. J. Shen L. Lehman L. H. Feng M. Ghassemi M. (2016). MIMIC-III, a freely accessible critical care database. Sci. Data 3, 160035. 10.1038/sdata.2016.35 27219127 PMC4878278

[B19] Johnson K. B. Wei W. Weeraratne D. Frisse M. E. Misulis K. Rhee K. (2021). Precision medicine, AI, and the future of personalized health care. Clin. Transl. Sci. 14, 86–93. 10.1111/cts.12884 32961010 PMC7877825

[B20] Khraim F. M. Carey M. G. (2009). Predictors of pre-hospital delay among patients with acute myocardial infarction. Patient Educ. Couns. 75, 155–161. 10.1016/j.pec.2008.09.019 19036551

[B21] Knaus W. A. Wagner D. P. Draper E. A. Zimmerman J. E. Bergner M. Bastos P. G. (1991). The Apache III prognostic system. Risk prediction of hospital mortality for critically ill hospitalized adults. Chest 100, 1619–1636. 10.1378/chest.100.6.1619 1959406

[B22] Lambden S. Laterre P. F. Levy M. M. Francois B. (2019). The SOFA score—development, utility and challenges of accurate assessment in clinical trials. Crit. Care 23, 374. 10.1186/s13054-019-2663-7 31775846 PMC6880479

[B23] Latif J. Xiao C. Tu S. Rehman S. U. Imran A. Bilal A. (2020). Implementation and use of disease diagnosis systems for electronic medical records based on machine learning: a complete review. IEEE Access 8, 150489–150513. 10.1109/ACCESS.2020.3016782

[B24] Le Gall J. Lemeshow S. Saulnier F. (1993). A new Simplified Acute Physiology Score (SAPS II) based on a European/North American multicenter study. JAMA 270, 2957–2963. 10.1001/jama.270.24.2957 8254858

[B25] Lee J. Lee J. Cho S. Song J. Lee M. Kim S. H. (2021). Development of decision support software for deep learning-based automated retinal disease screening using relatively limited fundus photograph data. Electronics 10, 163. 10.3390/electronics10020163

[B26] Liu Z. Zuo M. J. Xu H. (2012). “Parameter selection for Gaussian radial basis function in support vector machine classification,” in 2012 international conference on quality, reliability, risk, maintenance, and safety engineering (Chengdu, China: IEEE), 576–581. 10.1109/ICQR2MSE.2012.6246300

[B27] Mal K. Awan I. Shaukat F. (2019). Evaluation of risk factors associated with reinfarction: a multicenter observational study. Cureus 11, e6063. 10.7759/cureus.6063 31827993 PMC6890158

[B28] Manino E. Tran-Thanh L. Jennings N. R. (2019). On the efficiency of data collection for multiple naïve Bayes classifiers. Artif. Intell. 275, 356–378. 10.1016/j.artint.2019.06.010

[B29] Marshall J. C. (2020). Measuring organ dysfunction. Med. Klin. Intensivmed. Notfmed. 115, 15–20. 10.1007/s00063-020-00660-9 32077983

[B30] Moreno R. P. Nassar Júnior A. P. (2017). Is Apache II a useful tool for clinical research? Rev. Bras. Ter. Intensiva 29, 264–267. 10.5935/0103-507X.20170046 29044301 PMC5632966

[B31] Myles A. J. Feudale R. N. Liu Y. Woody N. A. Brown S. D. (2004). An introduction to decision tree modeling. J. Chemom. 18, 275–285. 10.1002/cem.873

[B32] Nasimov R. Muminov B. Mirzahalilov S. Nasimova N. (2020). “A new approach to classifying myocardial infarction and cardiomyopathy using deep learning,” in 2020 international conference on information science and communications technologies (ICISCT), 1–5. 10.1109/ICISCT50599.2020.9351386

[B33] Noble W. S. (2006). What is a support vector machine? Nat. Biotechnol. 24, 1565–1567. 10.1038/nbt1206-1565 17160063

[B34] Nordenskjöld A. M. Lagerqvist B. Baron T. Jernberg T. Hadziosmanovic N. Reynolds H. R. (2019). Reinfarction in patients with myocardial infarction with nonobstructive coronary arteries (MINOCA): coronary findings and prognosis. Am. J. Med. 132, 335–346. 10.1016/j.amjmed.2018.10.007 30367850

[B35] Okamoto K. Yamamoto T. Santos L. H. O. Ohtera S. Sugiyama O. Yamamoto G. (2020). Detecting severe incidents from electronic medical records using machine learning methods. Stud. Health Technol. Inf. 270, 1247–1248. 10.3233/SHTI200385 32570602

[B36] Padierna L. C. Carpio M. Rojas-Domínguez A. Puga H. Fraire H. (2018). A novel formulation of orthogonal polynomial kernel functions for SVM classifiers: the gegenbauer family. Pattern Recognit. 84, 211–225. 10.1016/j.patcog.2018.07.010

[B37] Pathmanathan A. (2005). Significance of positive Stenotrophomonas maltophilia culture in acute respiratory tract infection. Eur. Respir. J. 25, 911–914. 10.1183/09031936.05.00096704 15863651

[B38] Reed G. W. Rossi J. E. Cannon C. P. (2017). Acute myocardial infarction. Lancet 389, 197–210. 10.1016/S0140-6736(16)30677-8 27502078

[B39] Roth G. A. Mensah G. A. Johnson C. O. Addolorato G. Ammirati E. Baddour L. M. (2020). Global burden of cardiovascular diseases and risk factors 1990–2019: Update from the GBD 2019 study. J. Am. Coll. Cardiol. 76 (25), 2982–3021. 10.1016/j.jacc.2020.11.010 33309175 PMC7755038

[B53] Sadaka F. EthmaneAbouElMaali C. Cytron M. A. Fowler K. Javaux V. M. O’Brien J. (2017). Predicting mortality of patients with sepsis: A comparison of APACHE II and APACHE III scoring systems. J. Clin. Med. Res. 9, 907–910. 10.14740/jocmr3083w 29038667 PMC5633090

[B40] Shawe-Taylor J. Bartlett P. L. Williamson R. C. Anthony M. (1998). Structural risk minimization over data-dependent hierarchies. IEEE Trans. Inf. Theory 44, 1926–1940. 10.1109/18.705570

[B51] Scherpf M. Gräßer F. Malberg H. Zaunseder S. (2019). Predicting sepsis with a recurrent neural network using the MIMIC III database Comput. Biol. Med. 113, 103395. 10.1016/j.compbiomed.2019.103395 31480008

[B41] Singh K. Mayo P. (2018). Transthoracic echocardiography and mortality in sepsis: are we there yet? Intensive Care Med. 44, 1342–1343. 10.1007/s00134-018-5261-2 29943086

[B42] Than M. P. Pickering J. W. Sandoval Y. Shah A. S. V. Tsanas A. Apple F. S. (2019). Machine learning to predict the likelihood of acute myocardial infarction. Circulation 140, 899–909. 10.1161/CIRCULATIONAHA.119.041980 PMC6749969 31416346

[B43] Tharwat A. (2019). Parameter investigation of support vector machine classifier with kernel functions. Knowl. Inf. Syst. 61, 1269–1302. 10.1007/s10115-019-01335-4

[B44] Vapnik V. N. (2000). The nature of statistical learning theory. New York, NY: Springer. 10.1007/978-1-4757-3264-1

[B45] Vos T. Lim S. S. Abbafati C. (2020). Global burden of 369 diseases and injuries in 204 countries and territories, 1990-2019: a systematic analysis for the global burden of disease study 2019. Lancet 396, 1204–1222. 10.1016/S0140-6736(20)30925-9 33069326 PMC7567026

[B46] Wang S. McDermott M. B. A. Chauhan G. Ghassemi M. Hughes M. C. Naumann T. (2020). “MIMIC-extract: a data extraction, preprocessing, and representation pipeline for MIMIC-III,” in Proceedings of the ACM conference on health, inference, and learning (Toronto, Ontario: Canada: ACM), 222–235. 10.1145/3368555.3384469

[B47] Zhang L. Huang T. Xu F. Li S. Zheng S. Lyu J. (2022). Prediction of prognosis in elderly patients with sepsis based on machine learning (random survival forest). BMC Emerg. Med. 22, 26. 10.1186/s12873-022-00582-z 35148680 PMC8832779

[B48] Zhang M.-L. Zhou Z.-H. (2007). ML-KNN: a lazy learning approach to multi-label learning. Pattern Recognit. 40, 2038–2048. 10.1016/j.patcog.2006.12.019

[B49] Zhao Y. Xiong J. Hou Y. Zhu M. Lu Y. Xu Y. (2020). Early detection of ST-segment elevated myocardial infarction by artificial intelligence with 12-lead electrocardiogram. Int. J. Cardiol. 317, 223–230. 10.1016/j.ijcard.2020.04.089 32376417

